# Factors associated with outcomes in congenital duodenal obstruction: population-based study

**DOI:** 10.1093/bjs/znad040

**Published:** 2023-03-02

**Authors:** George S Bethell, Anna-May Long, Marian Knight, Nigel J Hall, BAPS-CASS, Abigail Jones, Adil Aslam, Alan Mortell, Amanda McCabe, Andrew Ross, Anna Harris, Anne Lawson, Arun Kelay, Aruna Abhyankar, Ashok Rajimwale, Atif Saeed, Bala Eradi, Baqer Sharif, Brian MacCormack, Caroline Pardy, Catherine Ridd, Ceri Jones, Ceri Jones, Chris Driver, Chris Parsons, Chun-Sui Kwok, Clare Rees, Clare Skerritt, Dan Aronson, David Marshall, Dawn Deacy, Debasish Banerjee, Diane De Caluwe, Dorothy Kufeji, Eleri Cusick, Elizabeth O’Connor, Georgina Bough, Govind Murthi, Hetal Patel, Ian Jones, Ian Sugarman, Ike Njere, Ingo Jester, Jonathan Durell, Kevin Cao, Khalid Elmalik, Lucinda Tullie, Madhavi Kakade, Maryam Haneef, Melania Matcovici, Michael Dawrant, Michelle Horridue, Miguel Soares-Oliveira, Miriam Doyle, Mohamed Shalaby, Morven Allan, Oliver Burdell, Paul Charlesworth, Paul Johnson, Richard Hill, Rosie Cresner, Ross Craigie, Samir Gupta, Sandeep Motiwale, Sanja Besarovic, Saravanakumar Paramalingam, Sean Marven, Shailesh Patel, Shazia Sharif, Shehryer Naqvi, Simon Clarke, Simon Kenny, Stefano Giuliani, Susan Payne, Thanos Tyraskis, Thomas Tsang, Tim Bradnock, William Calvert, Yatin Patel

**Affiliations:** University Surgery Unit, Faculty of Medicine, University of Southampton, Southampton, UK; Nuffield Department of Population Health, National Perinatal Epidemiology Unit, Oxford, UK; Department of Paediatric Surgery, Cambridge University Hospitals, Cambridge, UK; Nuffield Department of Population Health, National Perinatal Epidemiology Unit, Oxford, UK; University Surgery Unit, Faculty of Medicine, University of Southampton, Southampton, UK; Nuffield Department of Population Health, National Perinatal Epidemiology Unit, Oxford, UK

## Introduction

Congenital duodenal obstruction (CDO) is a rare condition (1.5 in 10 000 livebirths) requiring surgical correction during early life. The authors^1^ recently reported a large population-based epidemiological study of infants born with CDO. This study identified variation in management, in particular, there was variation in the surgical procedure performed, in the use of transanastomotic tube (TAT) feeding, central venous catheter (CVC) placement, and parenteral nutrition (PN) following surgical repair. If unwarranted variation exists, then identifying practices associated with improved patient outcomes is important. The association between these different treatments and patient outcomes was explored in the present planned secondary analysis.

## Methods

This study was carried out using the British Association of Paediatric Surgeons Congenital Anomaly Surveillance System (BAPS-CASS) following a prespecified, publicly available protocol^2^. Full details are provided in *supplementary material*.

### Patient identification and data collection

Liveborn infants with CDO (including atresia, stenosis, duodenal web or annular pancreas) presenting before 44 completed weeks postconceptual age were identified prospectively over a 1-year interval from 1 March 2016 at all 28 specialist paediatric surgical centres in the UK. Case identification and data collection procedures have been described previously^1^.

### Outcomes

Main outcomes defined *a priori* in the protocol were time to achieve full enteral feeds, use and duration of PN, number of CVCs used (including both peripherally inserted and centrally inserted catheters), CVC-related complications (infectious and non-infective), anastomotic complications, need for further surgery, duration of inpatient hospital stay, change in weight-for-age z score, and death. These outcomes reflect those reported in previous studies in this clinical field and also those felt to be most relevant to all stakeholders in CDO^3,4^. This includes parents who contributed to design of this study. A particular outcome of interest was whether TAT use reduces CVC use and CVC-associated complications such as sepsis. Outcomes were recorded at 28 days and 1 year after operative repair.

## Results

### Study population

Full details of the epidemiology, management, and outcomes of this cohort have been reported previously and salient details can be found in *supplementary material*^1,5,6^.

### Associated anomalies

Demographic and clinical characteristics were similar in infants with (*Table S1*) and without an associated anomaly, except that infants born with an associated anomaly had lower birthweight (*Table S2*). Overall management was similar, with the exception of surgical procedure performed, and outcomes were similar, except that infants with associated anomalies had a longer hospital stay (median 14 *versus* 21 days; *P* = 0.003) (*Table S3*). Given these differences, the presence of associated anomalies was included in multivariable models.

### Repair type

Group characteristics and management were similar for infants having either duodenoduodenostomy (DD) or duodenojejunostomy (DJ), except that those who underwent DJ were more likely to have a postampullary obstruction (71 *versus* 36 per cent; *P* = 0.043) (*Table S4*). Those who underwent DD reached full enteral feeds sooner (12 *versus* 16 days; *P* = 0.025) but had a greater decrease in weight-for-age z score at 28 days (*Table 1*).

**Table 1:** Outcomes by modifiable management strategies

	Repair type			TAT use			PN use		
	DD (*n* = 78)	DJ (*n* = 15)	P*	Yes (*n* = 43)	No (*n* = 59)	P*	Yes (*n* = 90)	No (*n* = 12)	P*
Postoperative time to commencing enteral feeds (days), median (range)	4 (1–35)	5.5 (1–31)	0.113	2 (1–24)	5 (1–35)	<0.001	4 (1–35)	2 (1–4)	0.002
Postoperative time to full enteral feeds (days), median (range)	12 (2–40)	16 (8–44)	0.025	10.5 (2–39)	13 (4–44)	0.095	13 (5–44)	6 (2–27)	<0.001
Duration of PN (days), median (range)	9 (0–86)	12.5 (0–22)	0.310	5.5 (0–22)	12 (0–86)	<0.001	–	–	–
No. of CVCs used, (days), median (range)	1 (0–8)	1 (0–4)	0.651	1 (0–2)	1 (0–8)	0.021	1 (0–8)	0 (0–1)	<0.001
CVC-related complication	17 (22)	3 (20)	1†	7 (16)	14 (24)	0.459†	21 (23)	0 (0)	0.068†
Repeat abdominal surgery within 28 days	3 (3.8)	0 (0)	1†	2 (4.7)	1 (1.7)	0.572†	5 (5.6)	0 (0)	1†
Postoperative duration of inpatient stay (days), median (range)	21 (9–73)	18.5 (11–29)	0.403	21.5 (9–73)	19 (6–72)	0.421	21 (8–73)	13 (6–29)	0.144
Change in standardized weight from birth to 28 days (z score), median (range)	−0.67 (−2.34 to 0.92)	−0.13 (−1.84 to 1.11)	0.018	−0.76 (−1.84 to 0.42)	−0.57 (−2.34 to 1.11)	0.072	−0.94 (−3.03 to 0.82)	−1.08 (−1.75 to –0.19)	0.501
Standardized weight at 1 year (z score), median (range)	−0.48 (−2.55 to 1.57)	−1.08 (−2.09 to 0.19)	0.345	−0.56 (−2.08 to 1.57)	−0.87 (−2.55 to 1.44)	0.878	−0.77 (−2.55 to 1.57)	−0.26 (−1.32 to 0.20)	0.535

Values are *n* (%) unless otherwise indicated. Those with repair types other than duodenoduodenostomy (DD) and duodenojejunostomy (JJ) or not specified (9 patients) were excluded. TAT, transanastomotic tube; PN, parenteral nutrition; CVC, central venous catheter. *Mann–Whitney *U* test, except †χ^2^ or Fisher’s exact test.

### Transanastomotic tube placement

TAT use was associated with younger age at surgery (2 *versus* 4 days; *P* = 0.005) (*Table S4*). Those with a TAT were less likely to have a CVC inserted than those without a TAT, and less likely to have PN (77 *versus* 97 per cent; *P* = 0.004) (*Table S4*); however, of the 43 who had a TAT placed, 34 also had a CVC placed and 33 received PN. TAT use was associated with earlier commencement of enteral feeds and shorter duration of PN (*Table 1*).

### Parenteral nutrition use

Among infants without PN, TAT placement was more common (83 *versus* 37 per cent; *P* = 0.004) and CVCs were used less often (25 *versus* 98 per cent; *P* < 0.001) (*Table S4*). Those who did not receive PN commenced enteral feeds earlier (2 *versus* 4 days; *P* = 0.002), achieved full enteral feeds earlier (6 *versus* 13 days; *P* < 0.001), and had fewer CVCs (median 0 *versus* 1 line; *P* < 0.001) (*Table 1*).

### Multivariable analysis

In multivariable analysis (*Table 2*), TAT placement was significantly associated with duration of PN. Other outcomes were similar regardless of modifiable management strategy.

**Table 2:** Multivariable analysis of main outcomes

	DD *versus* DJ		TAT (yes *versus* no)	
	Effect size	*P*	Effect size	*P*
Postoperative time to full enteral feeds (days)	−4.5 (−9.9, 0.81)	0.095	−2.1 (−5.6, 1.6)	0.276
Duration of PN (days)	−1.8 (−6.2, 2.5)	0.397	−5.1 (−8.0, −2.2)	0.001
No. of CVCs used	−0.10 (−0.53, 0.34)	0.659	−0.18 (−0.50, 0.14)	0.257
CVC-related complications	0.82 (0.18, 3.69)*	0.796	0.93 (0.30, 2.85)*	0.896
Postoperative duration of inpatient stay (days)	8.6 (−1.6, 18.8)	0.098	−0.25 (−7.8, 7.3)	0.947
Standardized weight at 1 year (z score)	0.38 (−0.51, 1.26)	0.396	−0.07 (−0.65, 0.51)	0.815

Values in parentheses are 95% confidence intervals. Effect sizes are mean differences, except *OR. Multivariable regression analysis of key outcomes was adjusted for birthweight, age at repair, presence of associated anomalies, operative technique, and transanastomotic tube (TAT) placement. DD, duodenoduodenostomy; DJ, duodenojejunostomy; PN, parenteral nutrition; CVC, central venous catheter.

## Discussion

Having previously reported variation in the operative and postoperative management of infants with CDO^1^, this study aimed to investigate the association between variation in clinical management and important clinical outcomes. The rationale for the study was that, if it is possible to identify clinical features or management strategies associated with improved clinical outcomes, then either practice should be standardized or these interventions should be a focus for future research.

Among different surgical methods for CDO repair, the most common are DD and DJ. The decision may be dictated by surgeon preference or patient anatomy. Reassuringly, outcomes associated with these two approaches were similar.

TAT feeding is employed by some surgeons after surgery for CDO to allow early enteral feeding which cannot be achieved by oral or nasogastric feeds owing to an element of gastric dysmotility secondary to the effects of CDO on the proximal gastrointestinal tract *in utero*^7,8^. In this study, TAT placement was associated with reduced CVC use, reduced duration of PN, and earlier commencement of enteral feeds in univariable analysis; the association with reduced duration of PN persisted in multivariable analysis. Previous studies^4,8,9^ have also reported benefits of TAT feeding on these and other outcomes, including reduced time to commencing enteral feeding, shorter times to full enteral feeds, reduced PN requirement, fewer central lines, and reduced cost. Despite this existing literature, less than half the infants in this study received a TAT.

It is interesting to consider why TAT use was not associated with improvement across all outcomes previously reported. The association between TAT use and number of CVCs and CVC-related complications noted previously^8^ was not seen here, nor was there an association between TAT use and time to full enteral feeds^4,7,8,10^. This suggests that, even when a TAT is used, nutritional management is such that the full benefits are not realized. For example, when a TAT was used, most infants also received a CVC and PN; this appears counterintuitive to the proposed benefits of a TAT. Similarly, it is plausible that, when a TAT was used, there was less urgency to advance enteral feeds because the majority had a CVC and hence the option of using PN.

Data from this cohort do, however, support the hypothesis that avoidance of PN is beneficial; almost all outcomes were better in infants without PN than among those who received PN, including a trend towards fewer CVC complications (*Table 1*). Although CVC use is routine in neonatal care, it does not come without risks, including death^11,12^. Complications remain common (1 in 4 in this series) and exposure to complications should not be underestimated. In a large series^13^, one-quarter of lines were removed prematurely owing to complications, including confirmed septicaemia in around 8 per cent.

Overall, these data reveal no association between co-existing congenital anomalies or repair type and important clinical outcomes. However, TAT use was associated with reduced duration of PN. Further work should explore nutritional interventions in this population in more detail as these data suggest that the full benefits of some feeding practices are not being fully realized.

## Collaborators

Members of BAPS-CASS: Abigail Jones, Queen's Medical Centre, Adil Aslam, Addenbrooke's Hospital, Alan Mortell, The Children's University Hospital, Amanda McCabe, Edinburgh Royal Hospital for Sick Children, Andrew Ross, Chelsea and Westminster Hospital, Anna Harris, Edinburgh Royal Hospital for Sick Children, Anne Lawson, Royal Victoria Infirmary, Arun Kelay, King's College Hospital, Aruna Abhyankar, University Hospital of Wales, Ashok Rajimwale, Leicester Royal Infirmary, Atif Saeed, Addenbrooke’s Hospital, Bala Eradi, Leicester Royal Infirmary, Baqer Sharif, Birmingham Children’s Hospital, Brian MacCormack, Royal Belfast Hospital for Sick Children, Caroline Pardy, St George’s Hospital, Catherine Ridd, The Great North Children’s Hospital, Ceri Jones, John Radcliffe Hospital, Ceri Jones, Southampton General Hospital, Chris Driver, Royal Aberdeen Children’s Hospital, Chris Parsons, Royal London Hospital, Chun-Sui Kwok, John Radcliffe Hospital, Clare Rees, Great Ormond Street Hospital for Sick Children, Clare Skerritt, Evelina Children’s Hospital, Dan Aronson, University Hospital of Wales, David Marshall, Royal Belfast Hospital for Sick Children, Dawn Deacy, The Children’s University Hospital, Debasish Banerjee, Norfolk and Norwich University Hospital, Diane De Caluwe, Chelsea and Westminster Hospital, Dorothy Kufeji, Evelina Children’s Hospital, Eleri Cusick, Bristol Royal Hospital for Children, Elizabeth O’Connor, The Great North Children’s Hospital, Georgina Bough, Addenbrooke’s Hospital, Govind Murthi, Sheffield Children’s Hospital, Hetal Patel, Glasgow Royal Hospital for Sick Children, Ian Jones, University Hospital of Wales, Ian Sugarman, Leeds General Infirmary, Ike Njere, St George’s Hospital, Ingo Jester, Birmingham Children’s Hospital, Jonathan Durell, Southampton General Hospital, Kevin Cao, Royal Alexandra Children’s Hospital, Khalid Elmalik, Leicester Royal Infirmary, Lucinda Tullie, Southampton General Hospital, Madhavi Kakade, Leicester Royal Infirmary, Maryam Haneef, Alder Hey Children’s Hospital, Melania Matcovici, The Children’s University Hospital, Michael Dawrant, Leeds General Infirmary, Michelle Horridue, Sheffield Children’s Hospital, Miguel Soares-Oliveira, Addenbrooke’s Hospital, Miriam Doyle, The Children’s University Hospital, Mohamed Shalaby, Bristol Royal Hospital for Children, Morven Allan, King’s College Hospital, Oliver Burdell, Norfolk and Norwich University Hospital, Paul Charlesworth, Royal London Hospital, Paul Johnson, John Radcliffe Hospital, Richard Hill, Leicester Royal Infirmary, Rosie Cresner, Chelsea and Westminster Hospital, Ross Craigie, Royal Manchester Children’s Hospital, Samir Gupta, Great Ormond Street Hospital for Sick Children, Sandeep Motiwale, Queen’s Medical Centre, Sanja Besarovic, Hull Royal Infirmary, Saravanakumar Paramalingam, Royal Alexandra Children’s Hospital, Sean Marven, Sheffield Children’s Hospital, Shailesh Patel, King’s College Hospital, Shazia Sharif, Royal London Hospital, Shehryer Naqvi, Royal Alexandra Children’s Hospital, Simon Clarke, Chelsea and Westminster Hospital, Simon Kenny, Alder Hey Children’s Hospital, Stefano Giuliani, St George’s Hospital, Susan Payne, Sheffield Children’s Hospital, Thanos Tyraskis, King’s College Hospital, Thomas Tsang, Norfolk and Norwich University Hospital, Tim Bradnock, Glasgow Royal Hospital for Sick Children, William Calvert, Alder Hey Children’s Hospital, Yatin Patel, Royal Aberdeen Children’s Hospital.

## Supplementary Material

znad040_Supplementary_Data

## Data Availability

Study protocol and data collection forms are available at https://www.npeu.ox.ac.uk/baps-cass.
