# Supplementary material for: Factors associated with outcomes in congenital duodenal obstruction: population-based study
Source: Br J Surg. 2023 Mar 2;110(9):1053–6. doi: 10.1093/bjs/znad040 (PMC10416685; doi:10.1093/bjs/znad040)
Supplement: znad040_Supplementary_Data [file znad040_supplementary_data.docx]

**Factors associated with outcomes in congenital duodenal obstruction: a population based study**

George S Bethell^1^, Anna-May Long^2,3^, Marian Knight^2^, Nigel J Hall^1^, On behalf of BAPS-CASS^2^

^1^University Surgery Unit, Faculty of Medicine, University of Southampton, Southampton, United Kingdom.

^2^National Perinatal Epidemiology Unit, Oxford, United Kingdom.

^3^Department of Paediatric Surgery, Cambridge University Hospitals, Cambridge, UK

**Corresponding author.** Nigel Hall, Associate Professor of Paediatric Surgery, University Surgery Unit, University of Southampton, Southampton, United Kingdom, SO16 6YD. [n.j.hall@soton.ac.uk](mailto:n.j.hall@soton.ac.uk).

**Supplementary Materials - Index**

| **Supplementary Methods** |  |
| --- | --- |
| Data collection | *pag. 2* |
| Statistical analysis | *pag. 2* |
| **Supplementary Results** |  |
| Study population and case management | *pag. 3* |
| Overall outcomes | *pag. 3* |
| **Supplementary Tables** |  |
| Table S1 – Associated anomalies with CDO. | *pag. 5* |
| Table S2 – Group characteristics and management undertaken comparing infants with and without associated anomalies. | *pag. 6* |
| Table S3 – Outcomes comparing infants with and without associated anomalies. | *pag. 7* |
| Table S4 – Group characteristics and management of infants by modifiable management strategies | *pag. 8* |
|  |  |
| **References** | *pag. 10* |
|  |  |

**Supplementary Methods**

**Ethical approval and case definition**

Ethical approval was provided by the National Research Ethics Service (NRES) South Central- Oxford A committee with a reference of 12/SC/0416. Cases of duodenal occlusion or narrowing caused by congenital bands associated with malrotation, intestinal volvulus, duplication cyst or malignancy without an intrinsic duodenal abnormality were excluded.

**Data collection**

Following case identification with return of monthly reporting cards from each specialist paediatric surgical centre a data collection form was sent for each identified case and completed at day 28 and 1 year following surgical repair. Forms were returned and entered into a database at the National Perinatal Epidemiological Unit (NPEU), Oxford. Cases not meeting the case definition and duplications were excluded.

**Statistical analysis**

Statistical analysis used StataSE v15 (StataCorp LLC, Texas, USA). Differences in demographic and clinical factors were explored as well as outcomes between groups of infants in univariable analyses using Fisher’s exact test for categorical data, chi-squared for categorical data with more than a 2x2 comparison and Mann Whitney-U test for continuous data. A P value of less than 0.05 was considered statistically significant. Data are presented as median (range). We compared group characteristics and outcomes for the two most common repair types – duodenoduodenostomy and duodenojejunostomy due to low numbers undergoing other types of repair.

Multivariable analyses using linear regression analysis or logistic regression analysis, as appropriate, was performed to investigate associations between outcomes of interest and modifiable management strategies adjusting for factors statistically significantly associated with outcomes in univariable analysis. In these multivariable analyses we also adjusted for demographic or clinical factors that were different between groups on univariable analysis (birthweight and age-at-repair) as well a presence of associated anomaly since an associated anomaly was identified in a high proportion of cases and some of these may be anticipated to impact outcomes of interest. Use of PN was not investigated as a management strategy as only 12 infants did not receive PN. Since both death and re-operation were rare events we did not perform multivariable analyses for these outcomes. Although we had originally intended (in our study protocol) to analyse outcomes following either laparoscopic or open surgical repair of CDO, since the proportion of cases having an initially laparoscopic approach was extremely low (5%) we did not compare outcomes stratified by operative approach.

To calculate standardised weight and weight change the zanthro package for StataSE v15 was used to calculate weight-for-age z scores using UK WHO term and preterm growth reference charts. For infants with Down syndrome the Zemel 2015 weight-for-age growth chart was used instead.(1) The weight-for-age z score, also known as SD score, is a measure of the SD of weight from the mean value of a reference population matched for gestational age and sex.(2)

**Supplementary Results**

**Study population and case management**

There were 102 cases in the study period that underwent operative repair of CDO. Of these, 55 (54%) were male and the median gestational age at birth was 36.3 (25.6-42.3) weeks with a median birthweight of 2458 (800-4320) grams.

Seventy infants (69%) had an associated anomaly (Supplementary Table 1), the most frequent being cardiac (n=48), chromosomal (n=38), and abnormal intestinal rotation (n=22). The most common method of operative repair was duodenoduodenostomy (n=78, 76%), followed by duodenojejunostomy (n=15, 15%). Laparoscopic repair was attempted in five (5%) infants with conversion to laparotomy in one of these. A trans-anastomotic tube (TAT) was inserted in 43 (42%) infants at time of surgical correction for CDO. A CVC was used in 91(89%) and PN was used in 90 (88%) infants.

**Overall outcomes**

Survival at 28 days following surgical repair of CDO was 98% (100/102), the two deaths were for reasons unrelated to CDO. The median time to commence enteral feeds was 4 (1-35) days and median time to full enteral feeds was 12 (2-44) days. There were two anastomotic complications within 28 days of initial procedure and in total by 1 year, 5 infants had a repeat laparotomy for reasons related to their initial CDO. There were 23 CVC related complications in 21 (21%) infants. In those where PN was used the median duration of this was 11 (2-134) days. The median postoperative hospital stay was 20 (6-73) days.

**Supplementary Figures and Tables**

| Table S1 - Associated anomalies (n=70*) | n(%) |
| --- | --- |
| Associated cardiac anomaly | 48 (47) |
| Isolated PDA | 6 (6) |
| PDA with other structural cardiac anomaly | 19 (19) |
| VSD | 17(17) |
| PFO | 15(15) |
| ASD | 10(10) |
| AVSD | 4 (4) |
| Tetralogy of Fallot | 3 (3) |
| Coarctation of aorta/hypoplasia | 2 (2) |
| Other | 11 (11) |
| Annular pancreas | 13 (13) |
| Biliary tree anomaly | 1 (1) |
| Abnormal rotation | 22 (22) |
| Other gastrointestinal anomaly | 15 (15) |
| Anorectal malformation | 5 (5) |
| OA with TOF | 5 (5) |
| Isolated OA | 4 (4) |
| Meckel’s diverticulum | 2 (2) |
| Ileal atresia | 2 (2) |
| Cloaca anomaly | 1 (1) |
| Genetic/chromosomal anomaly | 38 (37) |
| Trisomy 21 | 33 (32) |
| Other | 5 (5) |
| Other structural anomalies | 16 (16) |
| Renal | 5 (5) |
| Limb | 3 (3) |
| Spine | 2 (2) |
| Other | 11 (11) |

**Table S1 – Associated anomalies with CDO.** PDA = patent ductus arteriosus, VSD = ventricular septal defect, PFO = patent foramen ovale, ASD = atrial septal defect, AVSD = atrioventricular septal defect, OA = oesophageal atresia and TOF = tracheoesophageal fistula. *Note infants may have multiple anomalies therefore figures add up to more than 100%.

| Table S2 – Group characteristics and management of infants with and without associated anomalies | | | | |
| --- | --- | --- | --- | --- |
|  | | **No associated anomalies (n=32)** | **Associated anomalies (n=70)** | **p** |
| Male, n (%) | | 19 (59) | 36 (51) | 0.52 |
| Gestational age at birth, weeks (range) | | 37.1 (27-41) | 36 (25.6-42.3) | 0.28 |
| Birthweight, grams (range) | | 2552.5 (1550-4320) | 2298.5 (800-4080) | 0.05* |
| Age at surgery, days (range) | | 4 (1-14) | 2 (0-75) | 0.17 |
| Atresia type, n (%) | I | 14 (44) | 25 (36) | 0.43 |
|  | II | 0 (0) | 5 (7.1) |  |
|  | III | 12 (38) | 25 (36) |  |
|  | Not reported or not identified | 6 (19) | 15 (21) |  |
| Site of obstruction, n (%) | Pre-ampullary | 10 (32) | 15 (21) | 0.10 |
|  | Post-ampullary | 16 (52) | 29 (41) |  |
|  | Not reported or not identified | 5 (16) | 26 (37) |  |
| Repair type, n (%) | Duodenoduodenostomy | 24 (75) | 54 (78) | **0.04** |
|  | Duodenojejunostomy | 3 (9.4) | 12 (17) |  |
|  | Membrane incision | 0 (0) | 1 (1.4) |  |
|  | Membrane resection | 4 (12.5) | 0 (0) |  |
|  | Duodenoplasty | 1 (3.1) | 2 (2.9) |  |
| TAT used, n (%) | | 14 (44) | 29 (41) | 0.83 |
| CVC used, n (%) | | 29 (91) | 62 (89) | 1 |
| PN used, n (%) | | 27 (84) | 63 (90) | 0.51 |

**Table S2 – Group characteristics and management undertaken comparing infants with and without associated anomalies.** TAT = trans-anastomotic tube, CVC = central venous catheter, PN = parenteral nutrition. * denotes figure rounded down and therefore not statistically significant.

| Table S3 – Outcomes comparing infants with and without associated anomalies | | | |
| --- | --- | --- | --- |
|  | **No associated anomalies (n=32)** | **Associated anomalies (n=70)** | **p** |
| Time to commencing enteral feeds post op, days (range) | 4 (1-14) | 4 (1-35) | 0.65 |
| Time to full enteral feeds post op, days (range) | 10 (5-39) | 12 (2-44) | 0.23 |
| PN duration, days (range) | 8 (0-22) | 11 (0-86) | 0.14 |
| Number of CVCs used, n (range) | 1 (0-7) | 1 (0-8) | 0.50 |
| CVC related complication, n (%) | 5 (15) | 16 (23) | 0.60 |
| Repeat abdominal surgery within 28 days, n (%) | 0 (0) | 3 (4.3) | 0.55 |
| Inpatient stay post op, days (range) | 14 (6-50) | 21 (9-73) | **0.003** |
| Change in standardised weight from birth to 28 days, z score (range) | -0.73 (-1.84-0.53) | -0.64 (-2.34-1.11) | 0.89 |
| Standardised weight at 1 year, z score (range) | -0.33 (-1.95-1.57) | -0.97 (-2.55-1.44) | 0.35 |

**Table S3 – Outcomes comparing infants with and without associated anomalies.** PICC = peripherally inserted central catheter, CVC = central venous catheter, PN = parenteral nutrition.

| Table S4 – Group characteristics and management of infants by modifiable management strategies | | | | | | | | | | |
| --- | --- | --- | --- | --- | --- | --- | --- | --- | --- | --- |
|  | | **DD (n=78)** | **DJ (n=15)** | **p** | **TAT (n=43)** | **No TAT (n=59)** | **p** | **PN (n=90)** | **No PN (n=12)** | **p** |
| Male, n (%) | | 41 (53) | 8 (53) | 1 | 22 (51) | 33 (56) | 0.69 | 49 (54) | 6 (50) | 1 |
| Gestational age at birth, weeks (range) | | 36.3 (25.6-41) | 35.4 (28.1-39.4) | 0.48 | 36.3 (27-39.9) | 36.3 (25.6-42.3) | 0.88 | 36.3 (25.6-42.3) | 36.2 (27-39) | 0.55 |
| Birthweight, grams (range) | | 2487.5 (830-43200 | 2290 (800-3568) | 0.14 | 2350 (800-4320) | 2500 (830-3755) | 0.78 | 2457.5 (800-4320) | 2430 (2076-3600) | 0.38 |
| Age at surgery, days (range) | | 3 (0-75) | 1 (0-5) | **0.03** | 2 (0-14) | 4 (0-75) | **0.005** | 3 (0-75) | 2 (0-6) | 0.28 |
| Associated anomalies, n (%) | | 54 (69) | 12 (80) | 0.54 | 29 (67) | 41 (70) | 0.83 | 63 (70) | 7 (58) | 0.51 |
| Atresia type, n (%) | I | 27 (34) | 4 (27) | 0.07 | 13 (30) | 26 (44) | 0.42 | 35 (39) | 4 (33) | 0.90 |
|  | II | 3 (3.8) | 2 (13) |  | 3 (7.0) | 2 (3.4) |  | 4 (4.4) | 1 (8.3) |  |
|  | III | 34 (44) | 3 (20) |  | 16 (37) | 21 (36) |  | 33 (37) | 4 (33) |  |
|  | Not reported or not identified | 14 (18) | 6 (40) |  | 11 (26) | 10 (17) |  | 18 (20) | 3 (25) |  |
| Site of obstruction, n (%) | Pre-ampullary | 21 (27) | 2 (14) | **0.04** | 12 (28) | 13 (22) | 0.44 | 21 (24) | 4 (33) | 0.66 |
|  | Post-ampullary | 28 (36) | 10 (71) |  | 16 (37) | 29 (50) |  | 41 (46) | 4 (33) |  |
|  | Not reported or not identified | 29 (37) | 2 (14) |  | 15 (35) | 16 (28) |  | 27 (30) | 4 (33) |  |
| Repair type, n (%) | DD | NA | NA | NA | 26 (86) | 42 (71) | 0.11 | 68 (76) | 10 (83) | 0.80 |
|  | DJ | NA | NA |  | 5 (12) | 10 (17) |  | 14 (16) | 1 (8.3) |  |
| TAT used, n (%) | | 36 (46) | 5 (33) | 0.41 | NA | NA | NA | 33 (37) | 10 (83) | **0.004** |
| CVC used, n (%) | | 69 (89) | 14 (93) | 1 | 34 (79) | 57 (97) | **0.008** | 88 (98) | 3 (25) | **<0.0001** |
| PN used, n (%) | | 68 (87) | 14 (93) | 0.68 | 33 (77) | 57 (97) | **0.004** | NA | NA | NA |

**Table S4 - Group characteristics and management undertaken by repair type (Duodenoduodenostomy versus Duodenojejunostomy), trans-anastomotic tube use and parenteral nutrition use.** Those with repair types other than duodenoduodenostomy and duodenojejunostomy) or not specified (n=9) are excluded. DD = duodenoduodenostomy, DJ = duodenojejunostomy, TAT = trans-anastomotic tube, CVC = central venous catheter, PN = parenteral nutrition.

**References**

1. Zemel BS, Pipan M, Stallings VA, Hall W, Schadt K, Freedman DS, et al. Growth Charts for Children With Down Syndrome in the United States. Pediatrics. 2015;136(5):e1204-11.

2. de Onis M, Onyango AW, Borghi E, Garza C, Yang H, Group WMGRS. Comparison of the World Health Organization (WHO) Child Growth Standards and the National Center for Health Statistics/WHO international growth reference: implications for child health programmes. Public Health Nutr. 2006;9(7):942-7.
